# Chondrocytes and stem cells in 3D-bioprinted structures create human cartilage *in vivo*

**DOI:** 10.1371/journal.pone.0189428

**Published:** 2017-12-13

**Authors:** Peter Apelgren, Matteo Amoroso, Anders Lindahl, Camilla Brantsing, Nicole Rotter, Paul Gatenholm, Lars Kölby

**Affiliations:** 1 Department of Plastic Surgery, Institute of Clinical Sciences, Sahlgrenska University Hospital, Sahlgrenska Academy, Göteborg, Sweden; 2 Department of Clinical Chemistry and Transfusion Medicine, Institute of Biomedicine, Sahlgrenska University Hospital, Sahlgrenska Academy, Göteborg, Sweden; 3 Department of Otorhinolaryngology, University Medical Centre Ulm, Ulm, Germany; 4 3D Bioprinting Centre, Department of Chemistry and Chemical Engineering, Chalmers University of Technology, Göteborg, Sweden; Pohang University of Science and Technology, REPUBLIC OF KOREA

## Abstract

Cartilage repair and replacement is a major challenge in plastic reconstructive surgery. The development of a process capable of creating a patient-specific cartilage framework would be a major breakthrough. Here, we described methods for creating human cartilage *in vivo* and quantitatively assessing the proliferative capacity and cartilage-formation ability in mono- and co-cultures of human chondrocytes and human mesenchymal stem cells in a three-dimensional (3D)-bioprinted hydrogel scaffold. The 3D-bioprinted constructs (5 × 5 × 1.2 mm) were produced using nanofibrillated cellulose and alginate in combination with human chondrocytes and human mesenchymal stem cells using a 3D-extrusion bioprinter. Immediately following bioprinting, the constructs were implanted subcutaneously on the back of 48 nude mice and explanted after 30 and 60 days, respectively, for morphological and immunohistochemical examination. During explantation, the constructs were easy to handle, and the majority had retained their macroscopic grid appearance. Constructs consisting of human nasal chondrocytes showed good proliferation ability, with 17.2% of the surface areas covered with proliferating chondrocytes after 60 days. In constructs comprising a mixture of chondrocytes and stem cells, an additional proliferative effect was observed involving chondrocyte production of glycosaminoglycans and type 2 collagen. This clinically highly relevant study revealed 3D bioprinting as a promising technology for the creation of human cartilage.

## Introduction

A major challenge in reconstructive plastic surgery is the repair or replacement of damaged or absent cartilaginous structures, such as the auricle or the nose.[1] Current surgical procedures have several drawbacks involving complications, such as infections, tissue necrosis, and pain. Furthermore, the final outcome of surgery is often less than perfect,[2, 3] with these procedures constituting time-consuming and typically multi-staged processes.[4, 5]

Three-dimensional (3D)-bioprinting technology is a new approach allowing regeneration of cartilaginous structures using autologous cells dispersed in a biocompatible supporting framework. The 3D shape of the bioprinted construct can be very precise, which is of major importance for the reconstruction of specific structures, such as the nose and auricle. Additionally, this technique precludes invasive harvesting procedures, such as those involving rib cartilage[6, 7]; however, obstacles remain to be addressed before this biofabrication technology can be used clinically. Foremost among these are those related to the safety and long-term stability of the regenerated cartilage.

Inkjet[8–10] and extrusion printing[11–13] are the most commonly used modalities in the printing process. All methods use a medium (i.e., bioink) capable of maintaining the 3D shape of the cell-containing print and that also ensures the integrity of the construct in relation to the surrounding tissues. Bioinks are composed mainly of different natural biopolymers, including collagen,[14] alginate,[15] or hyaluronic acid,[16] or synthetic polymers, such as polyethylene glycol.[17] These polymers allow the gel to maintain a very high water content (i.e., hydrogels) and also provide the cells a favorable micro-milieu.[18, 19] The main advantages of these hydrogels concern their biocompatibility and low cytotoxicity[20]; however, hydrogels also limit the printing resolution due to their innate viscous properties. In transplant situations, it is critical to be able to handle the construct. To overcome hydrogel fragility and also increase shape fidelity, efforts have been made to stabilize the constructs with gelatine[21] or a combination of nanofibrillated cellulose (NFC) and alginate. Such hydrogel combinations also exhibit excellent shear-thinning properties and allow the creation of an adequate environment, which is important for maintaining high levels of cell viability post-printing.[22, 23]

Differentiated chondrocytes are difficult to obtain due to limited sources and complicated harvesting procedures. Therefore, alternative cell sources have been tested. An efficient and safe way to promote chondrogenesis involves the use of multipotent mesenchymal stem cells (MSCs), which have the ability to differentiate into chondrocytes, given the right environmental factors.[24–27] They also pose a lower risk of teratoma or osteogenic transformation as compared with embryonic stem cells[28, 29]; however, the most important factor in increasing chondrocyte proliferation involves MSC promotion of trophic activity during matrix formation.[30] The mechanisms associated with this activity remain largely unknown, although previous studies showed that MSCs excrete chondrocyte-promoting growth factors, such as fibroblast growth factor-1 and transforming growth factor-β,[31, 32] whereas others reported that physical cell-cell contacts were the crucial factor related to chondrogenesis.[33, 34] Therefore, co-cultures appear to be superior to monocultures, with the most efficient mixture of MSCs and chondrocytes reported as a 4:1 ratio.[35–37] Following cell mixing and homogenous distribution in the hydrogel ink, construction by the 3D bioprinter in a layer-by-layer assembly subsequently occurs.

In this study, we described the creation of cartilage from human chondrocytes *in vivo*, as well as quantitation of the chondrogenic potential of the chondrocytes alone and in combination with MSCs in 3D-bioprinted constructs implanted into nude mice.

## Materials and methods

### Cells

Human bone marrow derived MSCs (hBM-MSCs) were obtained from a female donor (Rooster Bio, Frederick, MD, USA). Human nasal chondrocytes (hNCs) were harvested from a male donor undergoing nasoseptal reconstruction at the Department of Otorhinolaryngology of Ulm University Medical Centre (Ulm, Germany). The harvesting was approved by the Ethical Advisory Board at Ulm University, Ulm, Germany (Dnr 152/08). The hBM-MSCs were cultured using an hBM-MSC high-performance media kit (RoosterBio) at 37°C in a humidified atmosphere with 5% CO_2_, passaged after 4 days, and harvested for printing on day 8. The hNCs were cultured in Dulbecco’s modified Eagle medium/F-12 (Life Technologies, Waltham, MA, USA) supplemented with 10% fetal bovine serum (HyClone; GE Healthcare, South Logan, UT, USA) and 1% penicillin/streptomycin (HyClone; GE Healthcare) for 6 days before printing.

### Bioinks and 3D bioprinting

**Fig 1:**
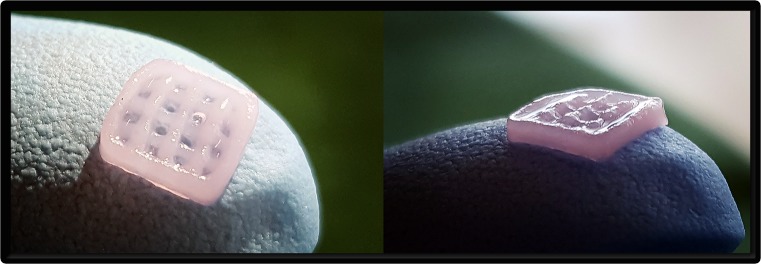
3D-bioprinted grid. Example of a cell-containing grid (5 × 5 × 1.2 mm) with a line spacing of 1.5 mm in three layers.

A 5 × 5 × 1.2-mm grid was printed using NFC bioink with 0.6-mm spacing (CELLINK AB, Gothenburg, Sweden) in an extrusion 3D bioprinter (INKREDIBLE; CELLINK AB) using laminated airflow (LAF bench; CELLINK AB) (Fig 1). The mixing procedure of the ink and cells was performed with a cell mixer (CELLINK AB) at a 11:1 ratio. The final cell density in all groups was 10 M cells/mL bioink. After printing, constructs were cross-linked with 100 mM CaCl_2_ for 5 min at 37°C, the cell-containing constructs were washed with their specific cell medium, and the blanks (without any cells) were washed with Hank’s balanced salt solution (HyClone; GE Healthcare). The printed constructs were then implanted into nude mice within 1 h.

### Animals

Forty eight 8-week old, female, nude Balb/C mice (Scanbur, Karlslunde, Denmark) were used. The animal experiment was performed at the core facility for experimental biomedicine at University of Gothenburg. European husbandry regulations were followed, as were those related to national and local regulations. After surgery, the animals were observed closely until they were wake and alert. After 4 hours, all mice were assessed again concerning alertness, signs of pain or bleeding. The morning after (approximately 15 hours after surgery), another check for complications were performed. During the whole study period, the well-being of the animals was assessed daily by an animal keeper and, if needed, a veterinarian.

The study was approved by the Ethics Committee for animal experiments at Sahlgrenska University Hospital/Gothenburg University, Göteborg, Sweden (Dnr 119–2015).

### Experimental design

The animals were divided into four groups, and the 3D-bioprinted constructs were surgically implanted in a subcutaneous pocket on the back of the mice. General anesthesia was induced by intraperitoneal injection of a mixture of ketamine (50 mg/mL) and medetomidine (1 mg/mL) at a 1.5:1 ratio. Each animal received 0.04 mL anesthetic solution per 20 g body weight. The skin pockets were closed with Vicryl Rapid (Ethicon, Sommerville, NJ, USA) and sealed with sterile wound tape. Each mouse then received 0.04 mL atipamezol (5 mg/mL) by intraperitoneal injection, to reverse the medetomidine effect. No antibiotics were used.

Each animal then carried the 3D-bioprinted construct containing one of the three different cell sets for 30 or 60 days. The fourth group carried cell-free NFC scaffolds (blanks) (Table 1). After 30 and 60 days, respectively, the animals were euthanized by cervical dislocation, and the constructs were harvested and fixated in 4% buffered formaldehyde supplemented with 20 mM CaCl_2_ overnight at 4°C and embedded in paraffin.

**Table 1:** Experimental design.

Group	Cell type	Composition	30 days	60 days
**1**	hNC	10 M cells/ml	n = 6	n = 6
**2**	Mix	20% hNC, 80% hBM-MSC^a^	n = 6	n = 6
**3**	hBM-MSC	10 M cells/ ml	n = 6	n = 6
**4**	Blank	NFC without cells	n = 6	n = 6

Experimental design, composition of the 3D constructs.

^a^10 M cells/ ml in total

### Morphological analysis

For morphological analysis and analysis of glycosaminoglycan (GAG) production, one core section (5 μm thickness) from every explanted construct was chosen (S1 Fig). Deparaffinized sections were stained with Alcian Blue and van Gieson. The sections were scanned using a Nikon Eclipse 90i epi-fluorescence microscope equipped with a Nikon DS-Fi2 color head camera and a NIS-Elements imaging software suite (vD4.10.02; Nikon Instruments, Melville, NY, USA). The image was imported into PhotoShop (Adobe Systems, San Hose, CA, USA), and the area of the section was determined. Every GAG-positive cell, with a concomitantly visible nucleus, in the sections was manually counted, and the GAG-positive cell-cluster areas (two or more GAG-positive cells, with stained extracellular matrices overlapping by 50% or more) were encircled (S2 Fig and S1 Appendix). The number of GAG-positive cells was presented as the number of cells ± standard deviation per mm^2^, and the sum of GAG-positive cell-cluster areas was related to the area of the section.

Additionally, all sections from group 1 and 2 were stained with Safranin-O to confirm the proteoglycan content within the GAG-positive areas.

### Immunohistochemical analysis

Deparaffinized sections were treated with 10 mM citrate buffer (pH 6) at 60°C overnight, followed by hyaluronidase treatment (Sigma-Aldrich, St. Louis, MO, USA) at 8000 U/mL in 0.1 M phosphate-buffered saline (PBS) for 60 min at 37°C. The sections were blocked with 10% goat serum (Thermo Fisher Scientific, Waltham, MA, USA) in 0.1 M PBS for 15 min at room temperature (RT). Thereafter, the sections were incubated with a monoclonal mouse anti-human type 2 collagen antibody IgG (1:150; Cat. No. 63171; Clone II-4C11; MP Biomedicals, Santa Ana, CA, USA) for 2 h at RT. The sections were again blocked with 10% goat serum in 0.1 M PBS for 15 min, followed by incubation with the secondary antibody [goat anti-mouse IgG (1:300) conjugated with AlexaFluor 546 (A11030; Thermo Fisher Scientific)] for 2 h at RT. Mounting solution was applied [ProLong Gold anti-fade mountant with 4′,6-diamidino-2-phenylindole (DAPI); Thermo Fisher Scientific], and the sections were stored overnight at 4°C.

Human cartilage derived from orthopedic knee surgery served as a positive control (Dnr S040-01). For the negative control, a monoclonal mouse IgG antibody (1:75; mouse IgG1 K isotype control purified; Cat. No. 14-4714-82; eBioscience; Thermo Fisher Scientific) was used.

Stained sections were analyzed using a Nikon Eclipse 90i epi-fluorescence microscope equipped with a Nikon ANDOR-Neo camera and the NIS-Elements imaging software suite (vD4.10.02; Nikon Instruments).

The methodology for the Ki-67 analysis is described in detail in the supplementary section (S2 Appendix).

### Fluorescent in situ hybridization (FISH) analysis

Paraffin sections (5 μm) were deparaffinized (3 x 10 min in dimethylbenzene), dehydrated in ethanol (2 mins each in 70%, 85% and 99,5%, respectively), and pretreated with sodium thiocyanate (1 mol/L) at 80°C for 30 min and RO-H_2_O for 3 min. Following protease treatment (dissolved in 0,9% NaCl) at 37°C for 45 min, the sections were again dehydrated in ethanol, probed with Vysis CEP X/Y (Cat. No. 07J22-050; Abbot Laboratories, Chicago, IL, USA), denatured at 85°C for 5 min, and hybridized overnight at 37°C (using Thermobrite, Leica Biosystems, Nussloch, Germany). After washing with SSC/0.05% Tween, the sections were counterstained with DAPI.

### Statistical analysis

An independent Student’s *t* test was used to compare the mean number of cells per mm^2^ and the mean percentage of the section area occupied by GAG-positive cell clusters in the hNC group (30 vs. 60 days), as well as in the mixed group (30 vs. 60 days). All statistical calculations were performed using SPSS (v22.0; IBM SPSS, Armonk, NY, USA). A *p* < 0.05 was considered statistically significant.

## Results

### Animals

In total, seven mice died during the first day of the study period due to anesthesiological complications. One mouse did not recover from surgery and died despite fluid resuscitation and repeated atipamezol doses. The other six were all wake and alert 4 hours after surgery but were found dead in their cages the morning after surgery. No signs of bleeding or any other adversities could be observed. The veterinarian at the core facility was consulted and concluded that the animals probably had died as a consequence of hypothermia.

Three of the deceased mice belonged to group 3, day 30, and three belonged to group 4, day 30. One animal in group 2 died, and analysis of an additional explant was not possible due to fractioning; therefore, the total number of analyzed constructs in group 2 at day 30 was four.

### Scaffolds

The NFC scaffolds, both with cells and cell-free blanks, were printed with high levels of printing fidelity and exhibited good printability and dimensional stability. All explanted constructs, except one in group 2 at day 30, retained their integrity and structural properties according to macroscopic analysis.

### Chondrogenesis and chondrocyte proliferation

**Fig 2:**
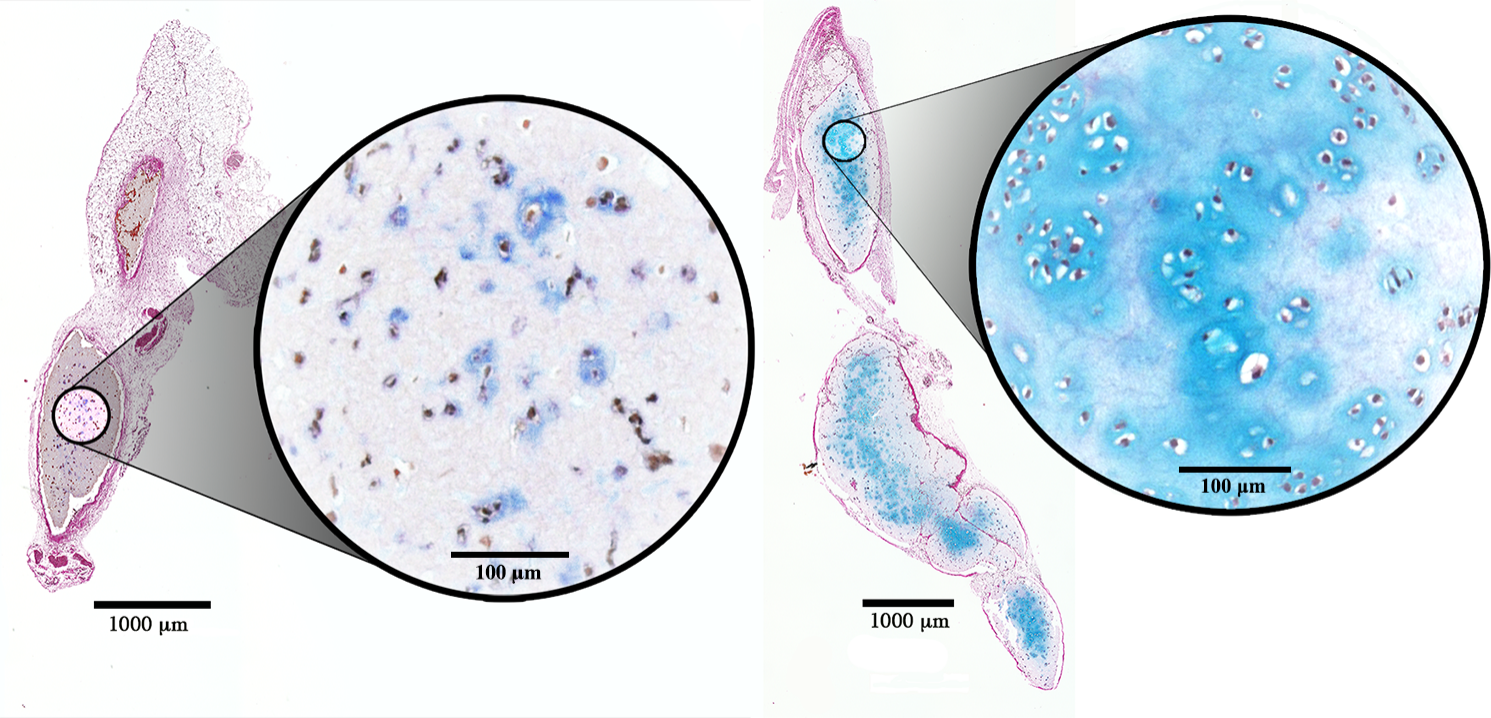
Chondrocyte proliferation. Histological sections of 3D-bioprinted constructs with hNCs at day 30 (*left*) and day 60 (*right*) after implantation. Alcian blue and van Gieson staining allow visualization of the glycosaminoglycans in the chondrocyte extracellular matrix. Progressive proliferation and cluster formation were observed after 60 days as compared to that observed after 30 days. Encircled magnifications show the cluster formations. Bars = 1000 μm and 100 μm (magnification).

Progressive chondrogenesis and proliferation of GAG-positive cells was observed in both the hNC group and the mixed group from day 30 to day 60. In group 1 (hNCs), there were 63.5 ± 86.2 (mean ± standard deviation) chondrocytes per mm^2^ after 30 days and 269.6 ± 78.8 chondrocytes per mm^2^ after 60 days (*p* = 0.001), with the percentage of the section area occupied by GAG-positive cell clusters increasing significantly from 3.0 ± 5.7% to 17.2 ± 7.7% (*p* = 0.004) at 30 and 60 days, respectively (Fig 2).

**Fig 3:**
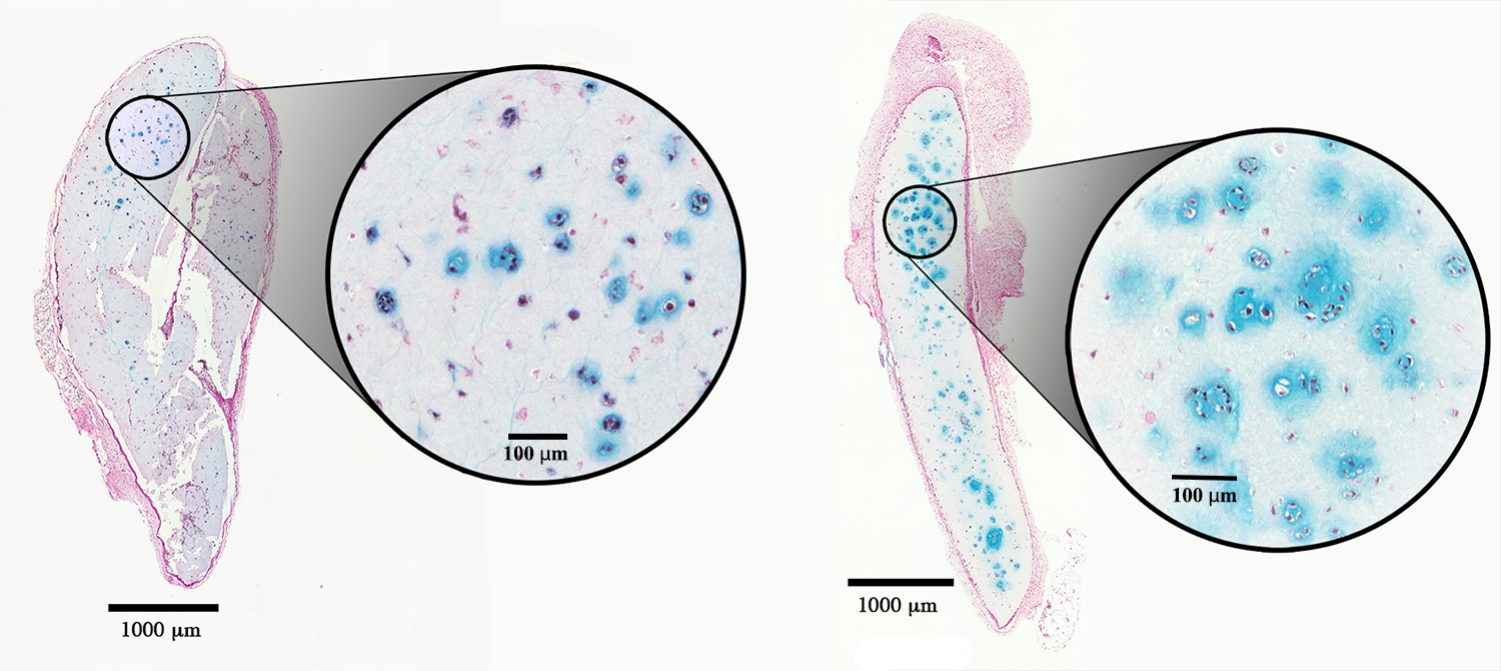
Chondrocyte proliferation. Histological sections of 3D-bioprinted constructs with hNCs mixed with hBM-MSCs at day 30 (*left*) and day 60 (*right*) after implantation. The glycosaminoglycans in the chondrocyte extracellular matrix were visualized by Alcian blue and van Gieson staining. Progressive proliferation and cluster formation seen after 60 days as compared with that observed after 30 days. Encircled magnifications show the cluster formations. Bars = 1000 μm and 100 μm (magnification).

In group 2 (mixed), there were 33.2 ± 39.1 chondrocytes per mm^2^ after 30 days and 166.8 ± 40.3 chondrocytes per mm^2^ after 60 days (*p* = 0.001), with the percentage of the section area occupied by GAG-positive cell clusters increasing significantly from 1.1 ± 1.4% to 12.4 ± 4.6% (*p* = 0.002) at 30 and 60 days, respectively (Fig 3).

**Fig 4:**
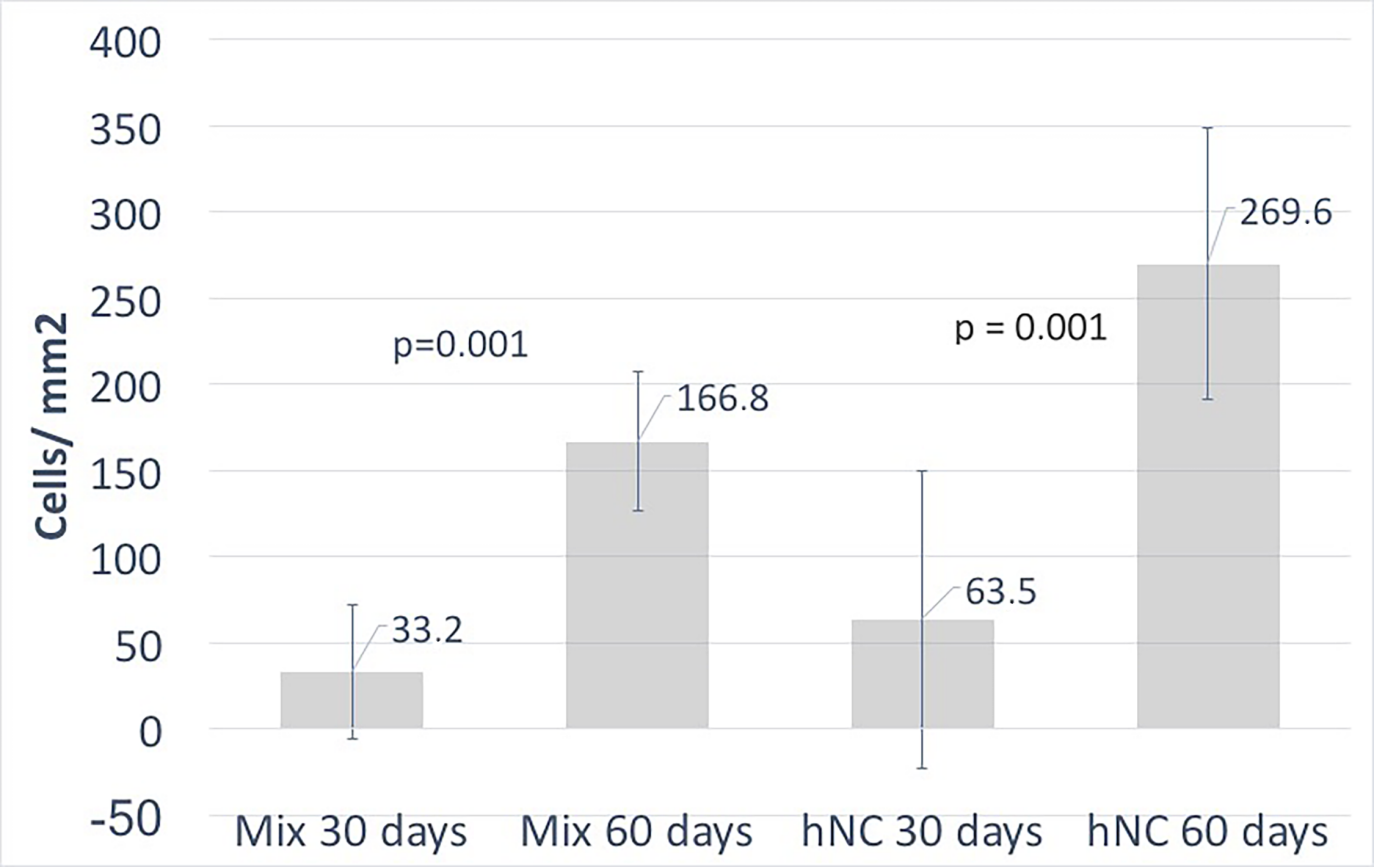
Number of chondrocytes per mm^2^. The proliferative increase in GAG-positive cells between days 30 and 60 was significant in both groups. The number of mature chondrocytes at the start of the experiment was 5-fold greater in the human nasal chondrocyte group as compared with the mixed group (10 M and 2 M, respectively), and the proliferative capacity was more pronounced in the mixed group.

**Fig 5:**
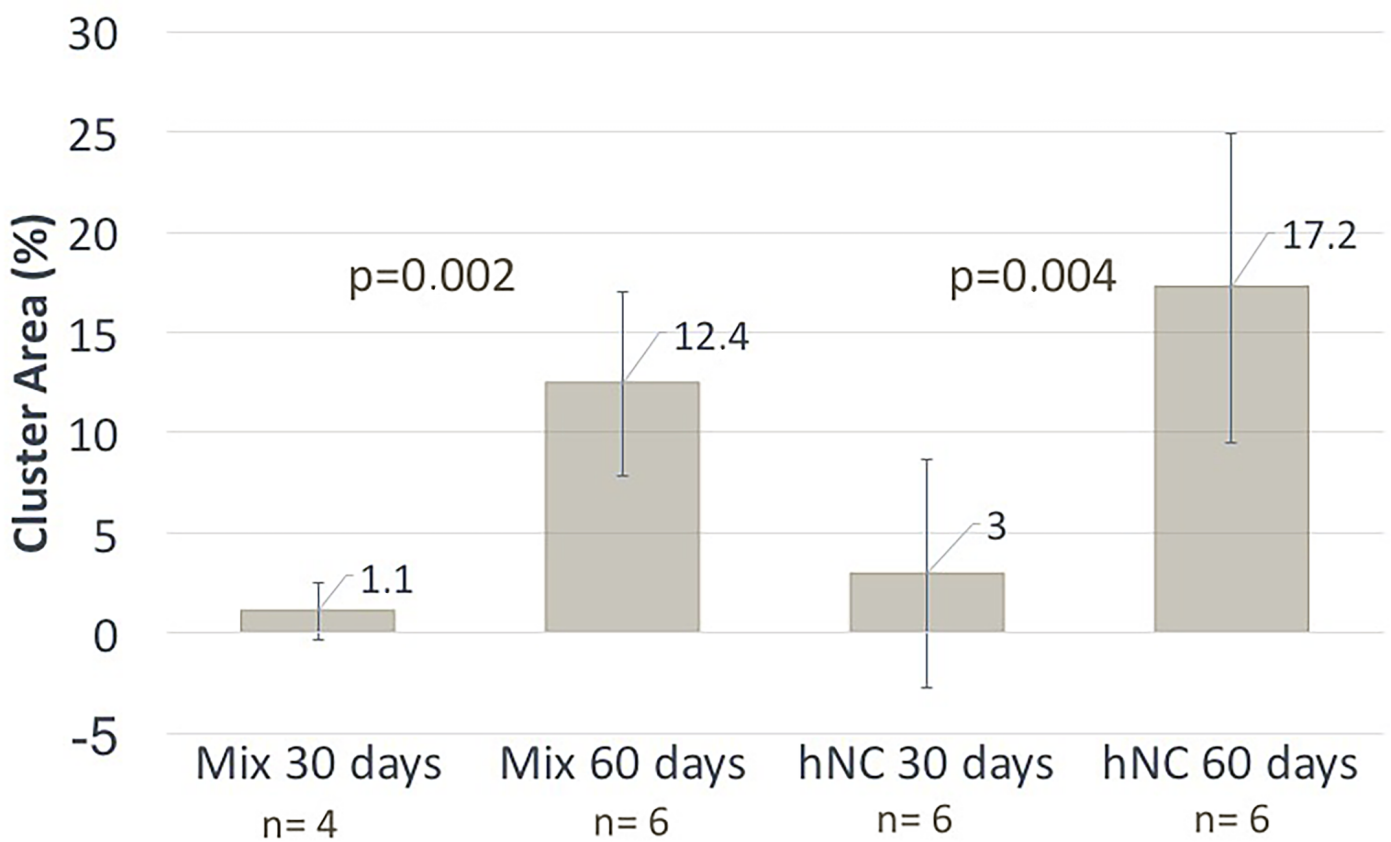
Cluster formation. Cluster area as a percentage of the entire area. Cluster formation represented a definitive sign of proliferation and resembled native cartilage.

Almost no GAG-positive chondrocytes were observed in group 3 (hBM-MSCs), and none were found in group 4 (the cell-free group). In addition to the proliferation reflected by the increased cell count over time in the cell-containing constructs, morphological analysis revealed that the GAG-positive chondrocytes formed clusters, representing a clear sign of proliferation. Microscopically, the cluster formations in the hNC group also showed good resemblance to native cartilage as opposed to the significantly smaller and more scattered clusters in the mixed group. These results are summarized in Fig 4 and Fig 5.

**Fig 6:**
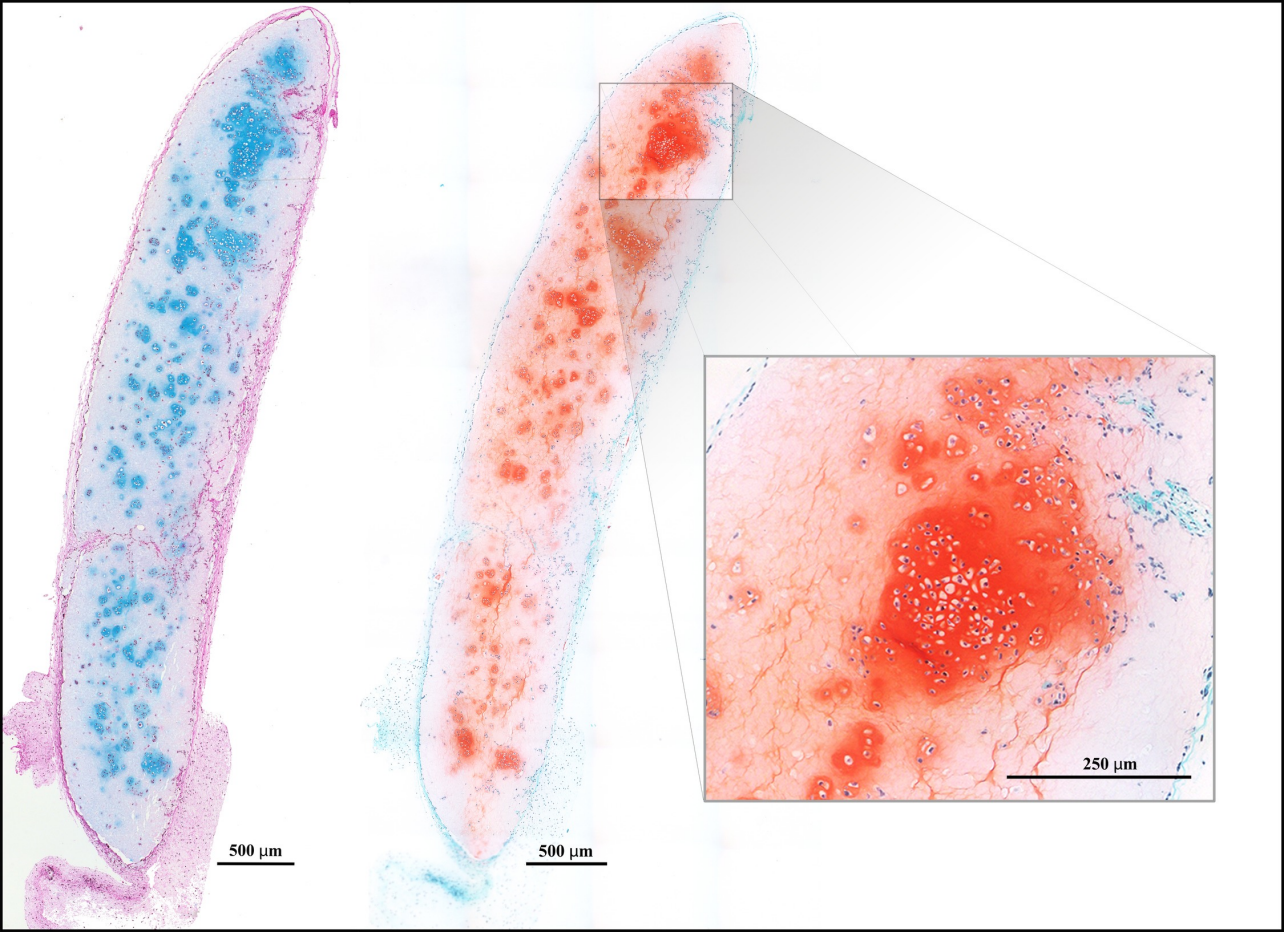
Safranin-O staining. Two consecutive sections from the mixed group (hNC/MSC) after 60 days. The GAG-positive extracellular matrix stains blue with Alcian blue and van Gieson (*left*). The proteoglycans in the extracellular matrix surrounding the chondrocytes are also visualized by Safranin-O staining (bright red) and nuclei are stained blue (*right*). The Safranin-O analysis confirm the results indicated by Alcian blue and van Gieson staining, i.e. similar areas are stained. Bars = 500 μm and 250 μm (magnification).

Additionally, Safranin-O staining corroborated the findings indicated by Alcian blue and van Gieson staining, i.e. the staining pattern of proteoglycans corresponded well with the GAG-positive areas. There are no signs of ossification. (Fig 6 and S3 Fig)

### FISH and immunohistochemical analyses

**Fig 7:**
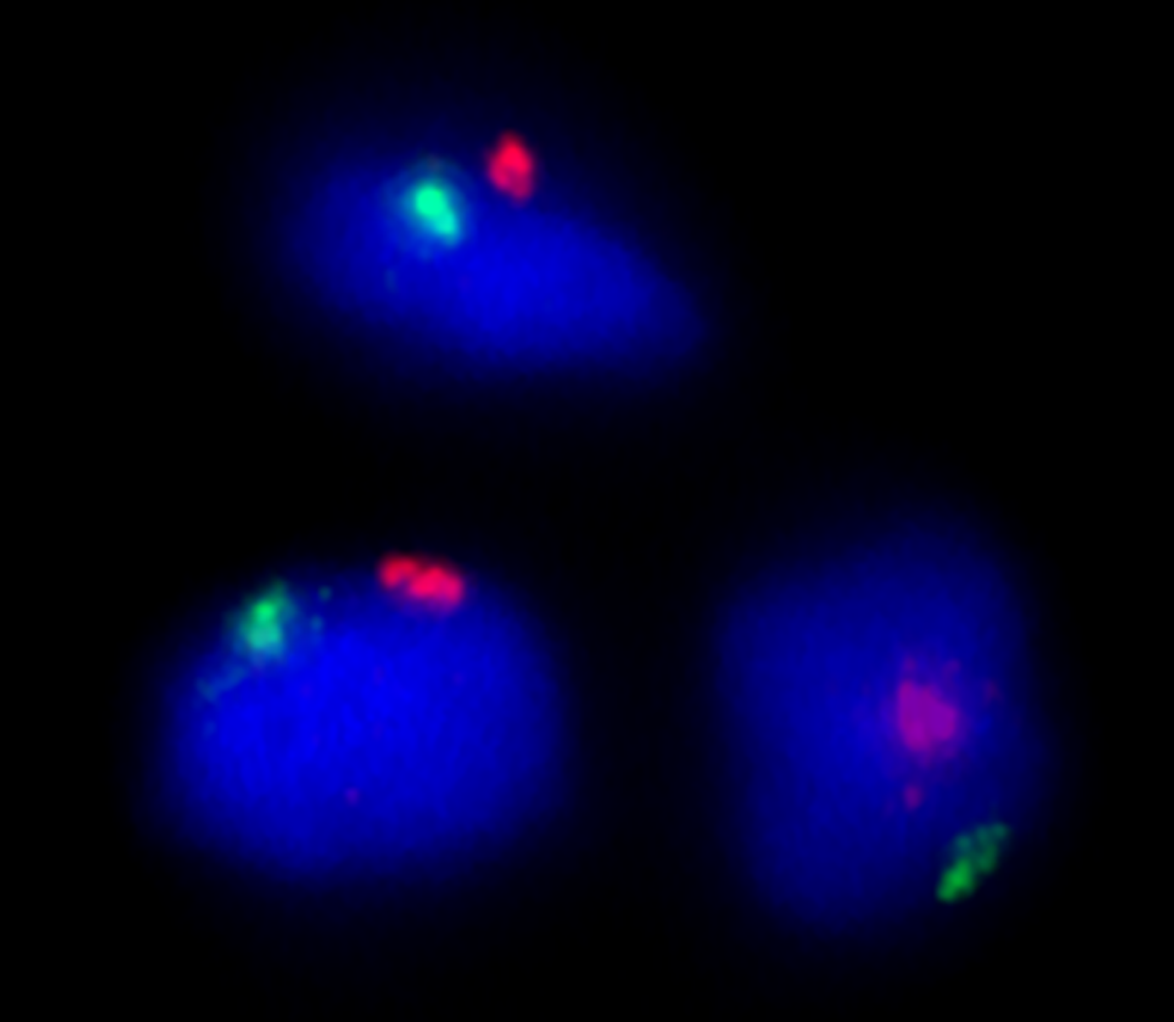
FISH analysis. FISH analysis to determine human chromosome X (green) and Y (red) confirmed that the proliferating cells in the chondrocyte clusters were of human origin. This analysis also concluded that the cells in the mixed group originated mainly from the male-derived chondrocytes (XY) and not the female-derived stem cells (XX).

**Fig 8:**
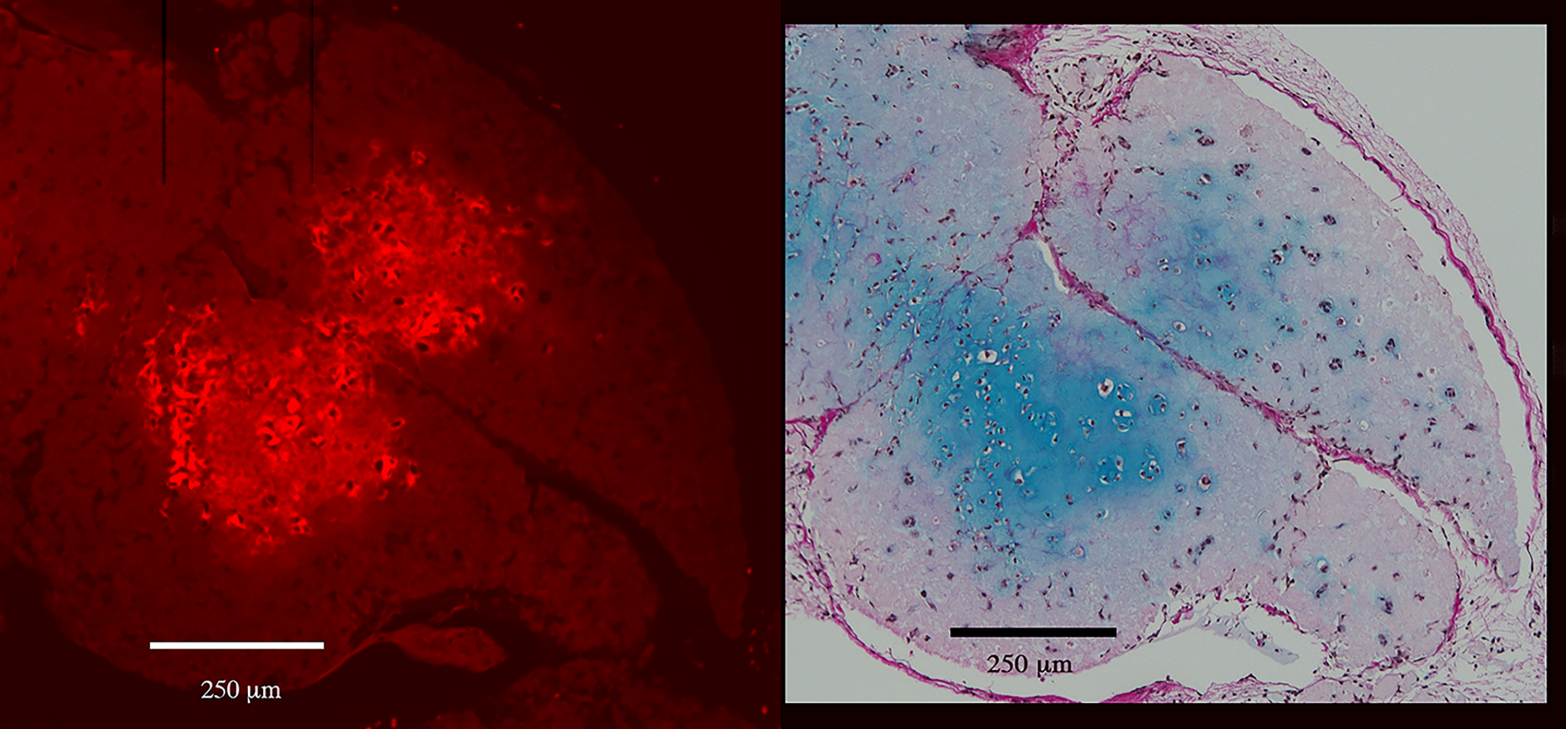
Immunohistochemical analysis. Immunohistochemical analysis of the type 2 collagen distribution (bright red, *left*) in the hNC constructs correlated well with the glycosaminoglycan production visualized by Alcian blue and van Gieson staining (*right*). Bars = 250 μm.

FISH analysis showed that the vast majority of the chondrocytes in the mixed group were positive for both human chromosome X and human chromosome Y, thereby confirming that the cells were of human origin, and that it was the chondrocytes (male) and not the stem cells (female) that had proliferated in the mixed group (Fig 7, S4 Fig and S3 Appendix). Additionally, results of immunohistochemical staining to analyze type 2 collagen distribution in the clusters correlated well with those related to GAG production observed by Alcian blue and van Gieson staining (Fig 8). These results indicated that the proliferated chondrocytes produced type 2 collagen, one of the extracellular proteins produced by native chondrocytes. Furthermore, the Ki-67 analysis confirmed proliferative activity at the moment of fixation (S5 Fig).

## Discussion

3D bioprinting has the potential to be of great value for several clinical applications in reconstructive surgery. The experimental diversity and complexity of 3D-bioprinting technology far exceeds its clinical relevance achieved in this field up to now. In this study, we described the *in vivo* application of cartilage bioprinting to a clinically relevant setting using adequate cells (human differentiated chondrocytes and human MSCs) and inert and mechanically stable bioink that allowed high-fidelity printing and creation of a favorable environment for the cells.

Our results revealed quantitative evidence of the proliferation of bioprinted chondrocytes, as well as data confirming presence of the boosting effect derived from co-culture with MSCs. Until now, there had been only qualitative descriptions of chondrocyte proliferation. In the present study, we verified findings reported in our pilot study by demonstrating proliferation of bioprinted, human chondrocytes resulting in the creation of cartilage *in vivo*. Mechanical testing of the constructs were made in the pilot study.[38]

Meticulous cell counting allowed accurate quantitative estimation of the proliferative capacity of the differentiated chondrocytes alone and in combination with MSCs. The manual counting procedure overcomes some major limitations associated with automatized methods, including problems with segmentation of histological images exhibiting large degrees of variability within image sets, underestimation due to multiple overlapping objects, and overestimation due to the inclusion of irrelevant objects.[39] Manual counting allows for more accurate quantification, because visual evaluation is performed on every cell, with only GAG-positive extracellular matrix surrounding cells with visible nuclei included. Additionally, the mixed group used in this study was determined by evaluating previous studies stating that a 20:80 ratio was the most favorable composition.[35–37]

The most important finding was the clear proliferative effect and cartilage formation observed over time. After 2 months, the *in vivo* morphology of the constructs resembled native cartilage, with a high percentage of the cross-sectional area occupied by GAG-positive cells. FISH and immunostaining for type 2 collagen confirmed both the human and the hyaline-cartilage origins of the new chondrocytes, with cluster formation of GAG-positive cells representing a definitive sign of proliferation. This was previously implied in our pilot study[38] and has now been quantitatively confirmed in this study using larger groups.

The highest density of GAG-positive cells was observed in constructs containing pure chondrocytes. To mimic clinically relevant situations, it is necessary to use fewer differentiated cells and replace them with stem cells, which are abundant. Here, the density of GAG-positive cells after 60 days in the mixed constructs was lower than in constructs harboring only chondrocytes. However, the original concentration of chondrocytes in the mixed constructs was only 20% that of the constructs harboring only chondrocytes. Despite this, the final GAG-positive cell count and GAG-positive area was only ~50% that observed in the mixed group and compared with pure hNC constructs. This indicated the influence of the stem cells on chondrocyte proliferation, and we cannot exclude the possibility that during a longer study period, a further increase in proliferation would be possible. This finding agreed with those of other studies showing that mixtures of chondrocytes and stem cells increased chondrocyte proliferation and facilitated cartilage formation.[35–37] Additionally, we confirmed that GAG-positive cells after 60 days were predominantly of chondrocyte origin and not derived from stem cell differentiation. As stated by the FDA Public Hearing on regulations of stem cells in 2016, the mechanism associated with the proliferative effect of stem cells on chondrocytes is related to paracrine signaling.[40]

The constructs created here were well integrated in mouse skin pockets and showed no signs of atypical cell growth or decomposition. However, the time span of 60 days was short and should be prolonged to allow appropriate evaluation of long-term stability, integration, and to ensure that no ossification or neoplasm development occurs. Previous studies have showed that the usage of mesenchymal stem cells, initially forms cartilage but eventually results in endochondral ossification.[41–43] However, these studies display several distinct differences compared to the present study. For example, the sophisticated cultivation procedures and the advanced maturation in vitro (e.g. chondrogenic priming) prior to the implantation as well as the usage of growth factors. In the present study, we used no growth factors and the cells were not cultivated or matured prior to the printing and implantation.

A limitation of the present study is that only two cell donors, one for stem cells and one for the chondrocytes, were used. This imply uncertainty regarding biological differences concerning the proliferative capacity which can be overcome by adding more donors and additional groups.

Several issues associated with the use of this method remain to be investigated and resolved, including the weight-bearing capabilities, shear-power resilience, elastic features, and degenerative resistance of the products. Furthermore, compliance with good clinical practices and the addressing of clinically important issues and logistics is required prior to the introduction of 3D-bioprinting techniques in the operating theater.[44–46] Future studies should focus on evaluation of other sources of multipotent stem cells, such as adipose-derived stem cells, to support chondrogenesis. These stem cells can be easily harvested, with some studies reporting their substantial proliferative potential.[47–51] Theoretically, adipose-derived stem cells could exert a boosting effect and similar cell-to-cell influence on chondrocytes as those observed in the presence of MSCs. Furthermore, to allow the creation of larger and more complex constructs, the critical issue of vascularization needs to be resolved. One technique used to overcome the diffusion limit of oxygen and nutrients (~200 μm) was described by Kang *et al*[52] and involved the incorporation of microchannels in the constructs, resulting in enhanced survival and cartilaginous tissue formation.

## Conclusions

In this study, we described the creation of viable cartilage *in vivo* using a 3D-bioprinted construct. The lineage with human nasal chondrocytes showed good proliferative ability in terms of cell number and cartilage-cluster formation. Furthermore, we observed that the addition of MSCs enhanced chondrocyte proliferation. This study described the establishment of a clinically relevant methodology that promotes the creation of custom-made, autologous cartilage in reconstructive surgery.

## Supporting information

S1 Fig Section of the blocks. The dimensions of the bioprinted constructs were 5 x 5 x 1.2 mm. The outermost millimeter on both sides was discarded in order to get representative sections. The remaining block (“core section”; 3 x 5 x 1.2 mm), were sliced in 5 μm sections. The three consecutive sections on each glass was evaluated and one of them chosen, based on staining quality and section coherency, for cell counting.
(TIF)

S2 Fig Definition of chondrocytes and clusters. The histological sections were analyzed regarding the number of chondrocytes (defined as a visible nucleus surrounded by extra cellular matrix (ECM) of glycosaminoglycans (GAGs) stained by Alcian blue and van Gieson (AvG)) and cluster area (defined as two or more nuclei surrounded by ECM of GAGs stained by AvG, which overlapped by 50% or more). (*A*) A single chondrocyte with a brown nucleus in lacunae, surrounded by a cloud of blue stained GAGs. (*B*) Two separate chondrocyte clusters containing two and three chondrocytes, respectively. The overlap is less than 50% and the two clusters is judged as separated. (*C*) A cluster containing five chondrocytes. (*D*) A large cluster containing 22 chondrocytes. (*E*) Metachromasia without any visible nucleus. The nucleus is probably located under or above the analyzed section and this cell is therefore not included. (*F*) A giant cluster containing 98 chondrocytes. (*G*) Two separated clusters containing 10 and 16 chondrocytes, respectively.
(TIF)

S3 Fig Safranin-O staining. An additional example of two consecutive sections from the hNC group after 60 days. Bar = 500 μm.
(TIF)

S4 Fig FISH analysis. One section from the hNC group (60 days) and one section from the mixed group (60 days) were analyzed with FISH. One hundred cells (out of 1740 and 930 in total, respectively) in each section were evaluated regarding human chromosomes X (green) and Y (orange). The first section (hNC; S3A) contained 100% male cells (i.e. XY) and the other section (mixed group hNC/MSC; S3B-D) contained 87 male cells and 13 female cells (i.e. XX). The protocol from the FISH assay is seen in S3E (2G = 2 green, 1G 1O = 1 green and 1 red). These results indicate that there was a vast majority of male cells in the mixed group, but also that some of the MSCs had survived.
(TIF)

S5 Fig Ki-67 analysis. Immunohistochemical analysis of Ki-67 in the mixed group at day 60, reveal proliferative activity at the moment of fixation. A similar appearance is observed in the hNC group (not shown). The green cloudy grains surrounding the DAPI stained chondrocyte nuclei (blue), represent Ki-67 positive cells (*A*). *B* show a section from another mixed construct with proliferating chondrocytes. Because of the significant amount of background auto-fluorescence from the biomaterial, the negative control from the same area as in *B* is shown in *C*. The Ki-67 and DAPI images are captured with 300 ms and 80 ms exposure time, respectively, with 40 x magnification and then merged. Except for cropping and added scale bars, no alterations of the images are made. Bars = 50 μm (*A*), 100 μm (*B* and *C*).
(TIF)

S1 Appendix Intra and inter observer variation. To test for the inter individual variability in the cell counting procedure, five randomly selected sections were counted manually by two evaluators. The composition of the sections was blinded and the intra individual variability were assessed by repeated counting of each section 3–6 times. The analysis of variance components for total cell count was done using the SAS Mixed Procedure (v9.4; SAS Institute Inc., Cary, NC, USA). The intra observer coefficient of variation for was 0.088 (95% CI 0.07–0.11) and the inter observer coefficient of variation was 0.087 (95% CI 0.04–3.9). To reduce the intra and inter observer variation, calibration between observers could be a good way to improve the counting method. The limited number of observers made the confidence interval for inter observer variation very wide and an increased number of observers would decrease this interval.
(PDF)

S2 Appendix Ki-67 analysis. Deparaffinised sections were treated with 10 mM citrate buffer (pH 6) at 90°C and permeabilized in 0.1% Triton X-100 (Sigma-Aldrich) in 0.1 M PBS for 15 min at RT. The sections were blocked with 0.1% Triton X-100, 2% bovine serum albumin (Sigma-Aldrich) and 0.7% glycin (Thermo Fischer Scientific) in 0.1M PBS for 30 min at RT. Thereafter, the sections were incubated with a monoclonal mouse anti-Ki67 antibody IgM (1:400; Cat. No. ab6526; Clone PP-67; Abcam, Cambridge, USA) over night at 4°C. The sections were again blocked with same solution as before, and then incubated with secondary antibody goat anti mouse IgM (1:300) conjugated with AlexaFluor 488 (A21042; Thermo Fisher Scientific) for 2h at RT. Mounting solution was applied (ProLong Gold antifade mountant with DAPI; Thermo Fisher Scientific), and the sections were stored overnight at 4°C. An old and, at the lab well-used, unlabeled section of rat skin served as positive control. For negative control the primary antibody was omitted. Stained sections were analyzed using a Nikon Eclipse 90i epi-fluorescence microscope equipped with a Nikon ANDOR-Neo camera and NIS-Elements imaging software suite (vD4.10.02; Nikon Instruments).
(PDF)

S3 Appendix The original FISH protocol with quantification data. (PDF)
