# Supplementary figures and images for: Chondrocytes and stem cells in 3D-bioprinted structures create human cartilage in vivo
Source: PLoS One. 2017 Dec 13;12(12):e0189428. doi: 10.1371/journal.pone.0189428 (PMC5728520; doi:10.1371/journal.pone.0189428)

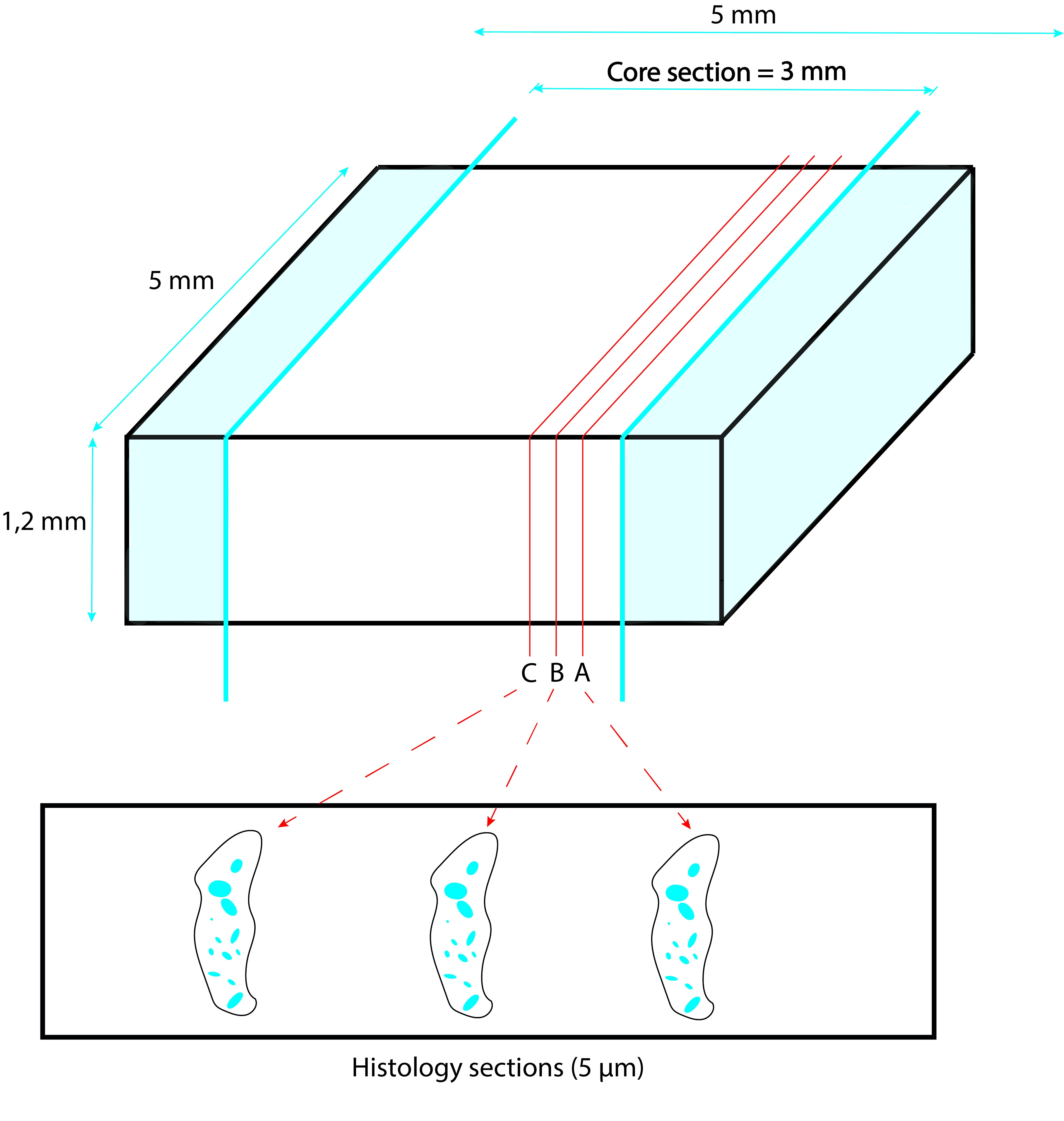

Supplement: S1 Fig — The dimensions of the bioprinted constructs were 5 x 5 x 1.2 mm. The outermost millimeter on both sides was discarded in order to get representative sections. The remaining block (“core section”; 3 x 5 x 1.2 mm), were sliced in 5 μm sections. The three consecutive sections on each glass was evaluated and one of them chosen, based on staining quality and section coherency, for cell counting. (TIF) [file pone.0189428.s001.tif]

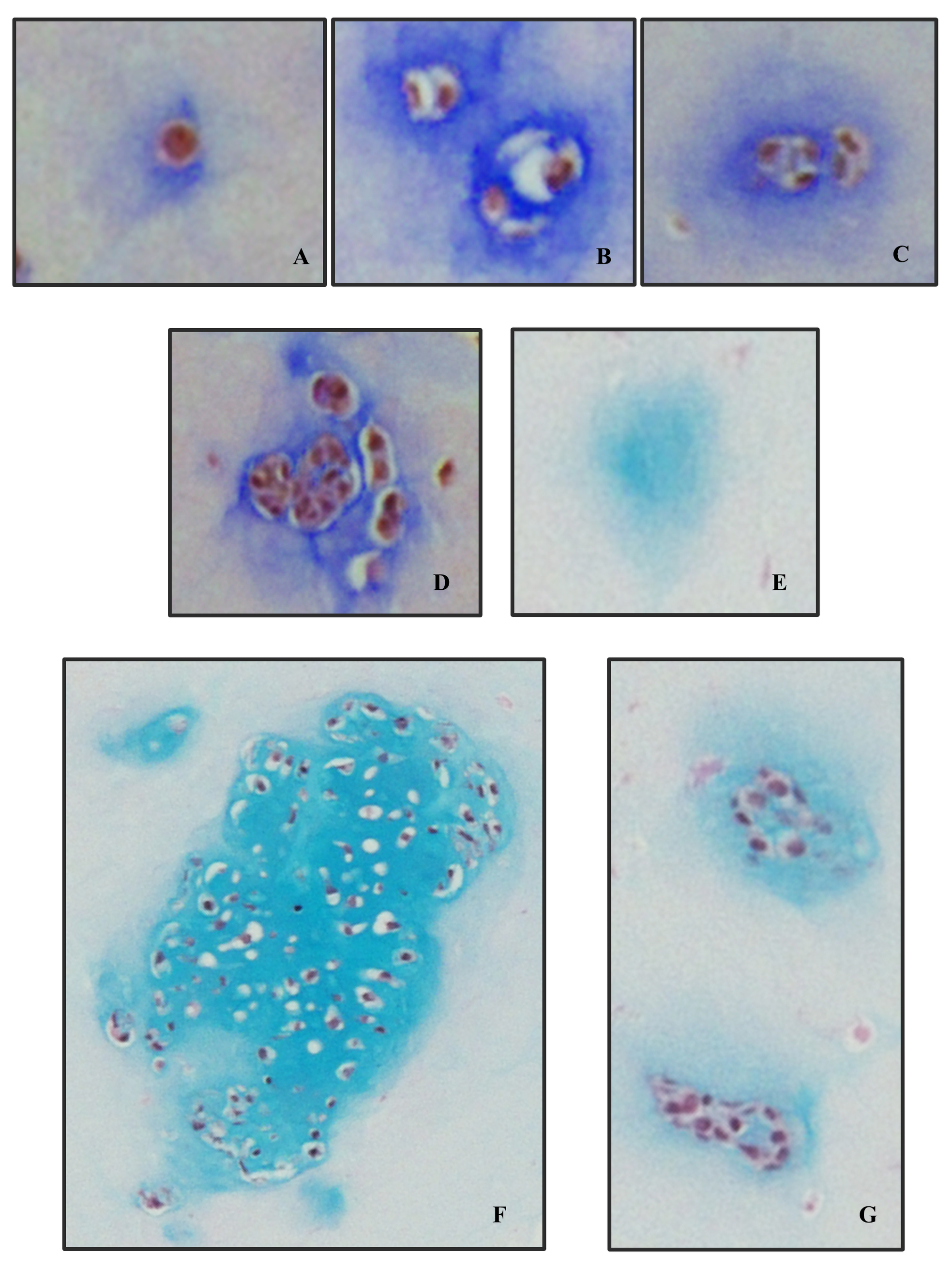

Supplement: S2 Fig — The histological sections were analyzed regarding the number of chondrocytes (defined as a visible nucleus surrounded by extra cellular matrix (ECM) of glycosaminoglycans (GAGs) stained by Alcian blue and van Gieson (AvG)) and cluster area (defined as two or more nuclei surrounded by ECM of GAGs stained by AvG, which overlapped by 50% or more). (A) A single chondrocyte with a brown nucleus in lacunae, surrounded by a cloud of blue stained GAGs. (B) Two separate chondrocyte clusters containing two and three chondrocytes, respectively. The overlap is less than 50% and the two clusters is judged as separated. (C) A cluster containing five chondrocytes. (D) A large cluster containing 22 chondrocytes. (E) Metachromasia without any visible nucleus. The nucleus is probably located under or above the analyzed section and this cell is therefore not included. (F) A giant cluster containing 98 chondrocytes. (G) Two separated clusters containing 10 and 16 chondrocytes, respectively. (TIF) [file pone.0189428.s002.tif]

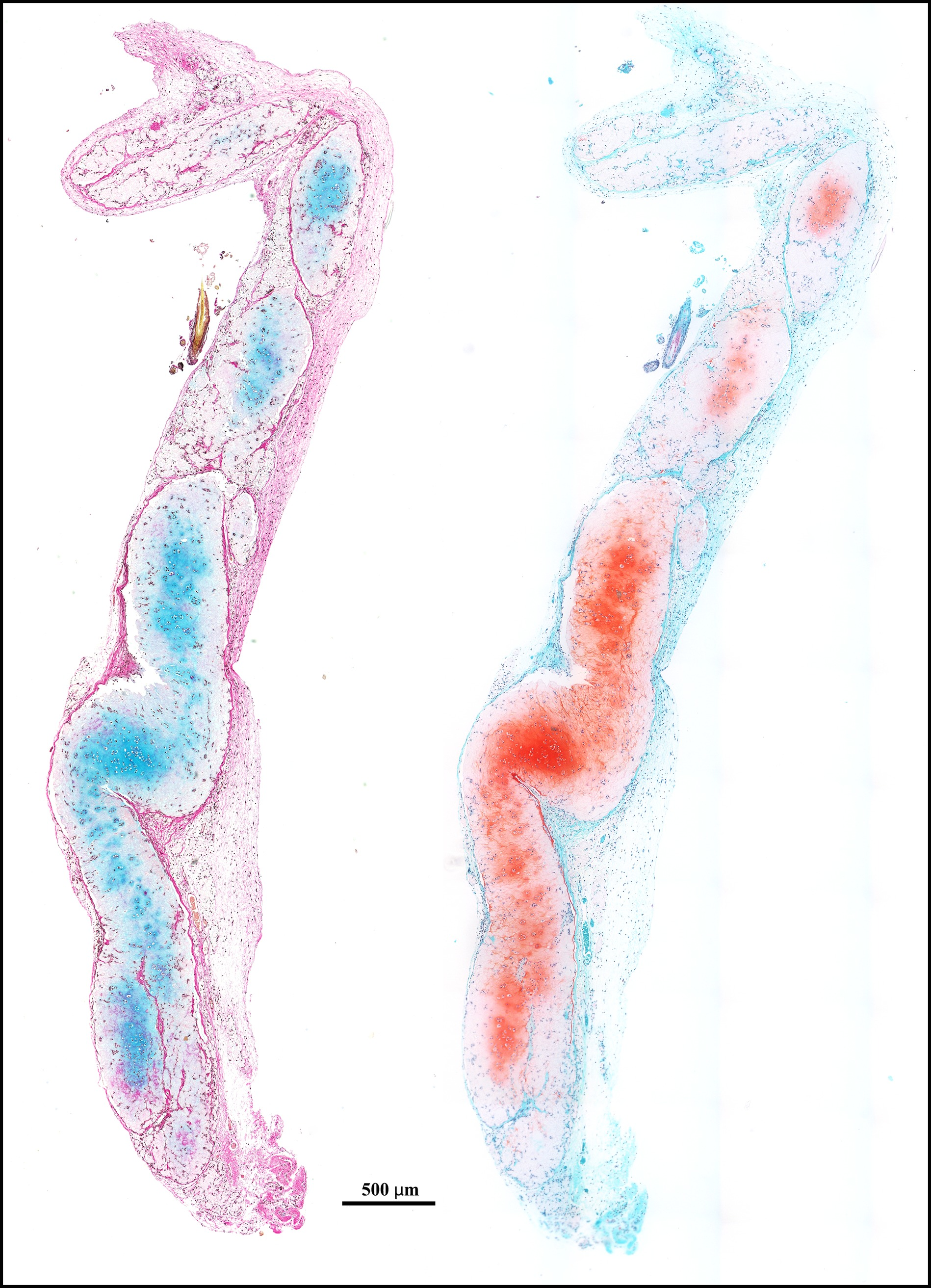

Supplement: S3 Fig — An additional example of two consecutive sections from the hNC group after 60 days. Bar = 500 μm. (TIF) [file pone.0189428.s003.tif]

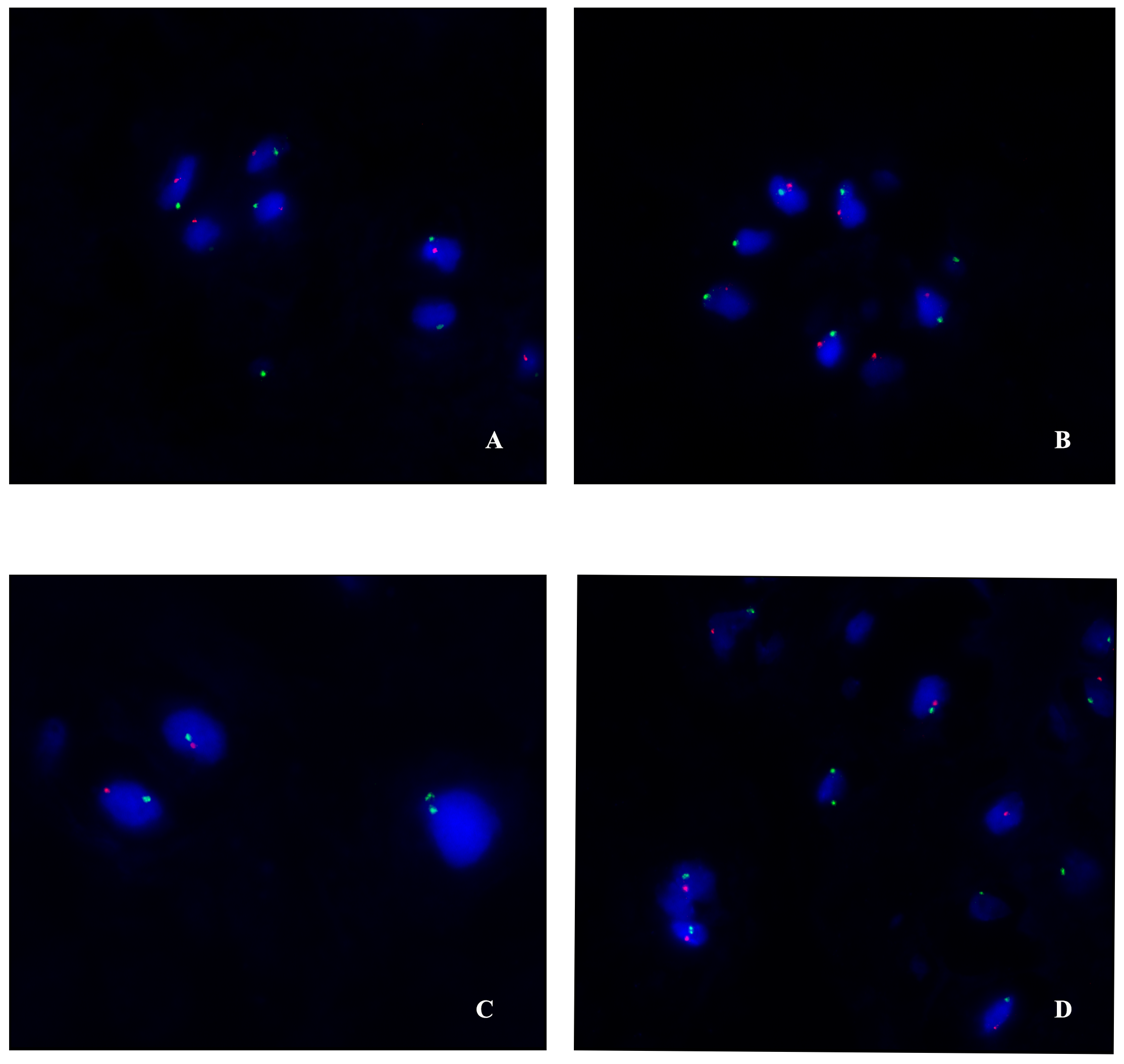

Supplement: S4 Fig — One section from the hNC group (60 days) and one section from the mixed group (60 days) were analyzed with FISH. One hundred cells (out of 1740 and 930 in total, respectively) in each section were evaluated regarding human chromosomes X (green) and Y (orange). The first section (hNC; S3A) contained 100% male cells (i.e. XY) and the other section (mixed group hNC/MSC; S3B-D) contained 87 male cells and 13 female cells (i.e. XX). The protocol from the FISH assay is seen in S3E (2G = 2 green, 1G 1O = 1 green and 1 red). These results indicate that there was a vast majority of male cells in the mixed group, but also that some of the MSCs had survived. (TIF) [file pone.0189428.s004.tif]

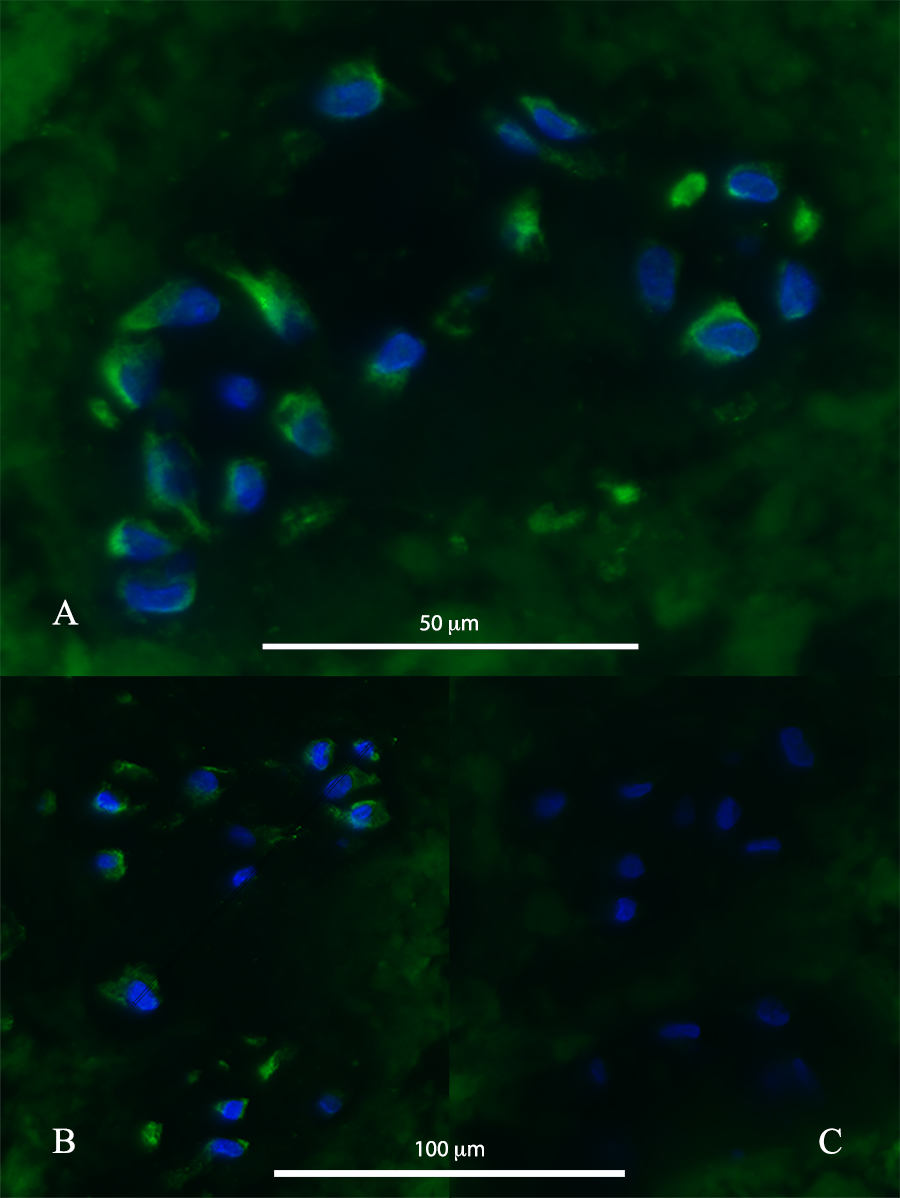

Supplement: S5 Fig — Immunohistochemical analysis of Ki-67 in the mixed group at day 60, reveal proliferative activity at the moment of fixation. A similar appearance is observed in the hNC group (not shown). The green cloudy grains surrounding the DAPI stained chondrocyte nuclei (blue), represent Ki-67 positive cells (A). B show a section from another mixed construct with proliferating chondrocytes. Because of the significant amount of background auto-fluorescence from the biomaterial, the negative control from the same area as in B is shown in C. The Ki-67 and DAPI images are captured with 300 ms and 80 ms exposure time, respectively, with 40 x magnification and then merged. Except for cropping and added scale bars, no alterations of the images are made. Bars = 50 μm (A), 100 μm (B and C). (TIF) [file pone.0189428.s005.tif]
