# Supplementary material for: Chondrocytes and stem cells in 3D-bioprinted structures create human cartilage in vivo
Source: PLoS One. 2017 Dec 13;12(12):e0189428. doi: 10.1371/journal.pone.0189428 (PMC5728520; doi:10.1371/journal.pone.0189428)
Supplement: S1 Appendix — To test for the inter individual variability in the cell counting procedure, five randomly selected sections were counted manually by two evaluators. The composition of the sections was blinded and the intra individual variability were assessed by repeated counting of each section 3–6 times. The analysis of variance components for total cell count was done using the SAS Mixed Procedure (v9.4; SAS Institute Inc., Cary, NC, USA). The intra observer coefficient of variation for was 0.088 (95% CI 0.07–0.11) and the inter observer coefficient of variation was 0.087 (95% CI 0.04–3.9). To reduce the intra and inter observer variation, calibration between observers could be a good way to improve the counting method. The limited number of observers made the confidence interval for inter observer variation very wide and an increased number of observers would decrease this interval. (PDF) [file pone.0189428.s006.pdf]

**S1 Appendix. Intra and inter observer variation.** To test for the inter individual variability in the cell counting procedure, five randomly selected sections were counted manually by two evaluators. The composition of the sections was blinded and the intra individual variability were assessed by repeated counting of each section 3-6 times. The analysis of variance components for total cell count was done using the SAS Mixed Procedure (v9.4; SAS Institute Inc., Cary, NC, USA). The intra observer coefficient of variation for was 0.088 (95% CI 0.07 - 0.11) and the inter observer coefficient of variation was 0.087 (95% CI 0.04 - 3.9). To reduce the intra and inter observer variation, calibration between observers could be a good way to improve the counting method. The limited number of observers made the confidence interval for inter observer variation very wide and an increased number of observers would decrease this interval.
