# Supplementary material for: Chondrocytes and stem cells in 3D-bioprinted structures create human cartilage in vivo
Source: PLoS One. 2017 Dec 13;12(12):e0189428. doi: 10.1371/journal.pone.0189428 (PMC5728520; doi:10.1371/journal.pone.0189428)
Supplement: S2 Appendix — Deparaffinised sections were treated with 10 mM citrate buffer (pH 6) at 90°C and permeabilized in 0.1% Triton X-100 (Sigma-Aldrich) in 0.1 M PBS for 15 min at RT. The sections were blocked with 0.1% Triton X-100, 2% bovine serum albumin (Sigma-Aldrich) and 0.7% glycin (Thermo Fischer Scientific) in 0.1M PBS for 30 min at RT. Thereafter, the sections were incubated with a monoclonal mouse anti-Ki67 antibody IgM (1:400; Cat. No. ab6526; Clone PP-67; Abcam, Cambridge, USA) over night at 4°C. The sections were again blocked with same solution as before, and then incubated with secondary antibody goat anti mouse IgM (1:300) conjugated with AlexaFluor 488 (A21042; Thermo Fisher Scientific) for 2h at RT. Mounting solution was applied (ProLong Gold antifade mountant with DAPI; Thermo Fisher Scientific), and the sections were stored overnight at 4°C. An old and, at the lab well-used, unlabeled section of rat skin served as positive control. For negative control the primary antibody was omitted. Stained sections were analyzed using a Nikon Eclipse 90i epi-fluorescence microscope equipped with a Nikon ANDOR-Neo camera and NIS-Elements imaging software suite (vD4.10.02; Nikon Instruments). (PDF) [file pone.0189428.s007.pdf]

S2 Appendix. Ki-67 analysis. Deparaffinised sections were treated with 10 mM citrate buffer (pH 6) at 90°C and permeabilized in 0.1% Triton X-100 (Sigma-Aldrich) in 0.1 M PBS for 15 min at RT. The sections were blocked with 0.1% Triton X-100, 2% bovine serum albumin (Sigma-Aldrich) and 0.7% glycin (Thermo Fischer Scientific) in 0.1M PBS for 30 min at RT. Thereafter, the sections were incubated with a monoclonal mouse anti-Ki67 antibody IgM (1:400; Cat. No. ab6526; Clone PP-67; Abcam, Cambridge, USA) over night at 4°C. The sections were again blocked with same solution as before, and then incubated with secondary antibody goat anti mouse IgM (1:300) conjugated with AlexaFluor 488 (A21042; Thermo Fisher Scientific) for 2h at RT. Mounting solution was applied (ProLong Gold antifade mountant with DAPI; Thermo Fisher Scientific), and the sections were stored overnight at 4°C. An old and, at the lab well-used, unlabeled section of rat skin served as positive control. For negative control the primary antibody was omitted. Stained sections were analyzed using a Nikon Eclipse 90i epi-fluorescence microscope equipped with a Nikon ANDOR-Neo camera and NIS-Elements imaging software suite (vD4.10.02; Nikon Instruments).
