# Supplementary material for: Chondrocytes and stem cells in 3D-bioprinted structures create human cartilage in vivo
Source: PLoS One. 2017 Dec 13;12(12):e0189428. doi: 10.1371/journal.pone.0189428 (PMC5728520; doi:10.1371/journal.pone.0189428)
Supplement: S3 Appendix — (PDF) [file pone.0189428.s008.pdf]

Arbetsprotokoll för FISH-analys — CEP X/Y Vysis

DXZ1 (Xp11.1-q11.1) (α satellit): Sp.Green **DYZ3 (Yp11.1-q11.1) (α satellit): Sp.Orange**

620161369 Pers.nr: 2016-09-22-U006
G4d60
57
Provt. datum: 16-09-22
Anders lindahl almedium

Diagnos:

Material: Smitt **PAD:**

Droppn. av preparat: **Sign:**

FISH analys dat: 160922 **Sign:** 10

Övrigt:

Analyserade interfaskärnor

Sign	2G	1G 1O					Total
10		100					100
**Summa**							
**Resultat %**							

Analyserade metafaser

Sign: Sign:

Koordinat	Resultat	Koordinat	Resultat	Koordinat	Resultat	Koordinat	Resultat

Fotograferade celler och metafaser:

Bild nr.	Koordinat	Resultat
2001	96,5-8,9	60
2		60
3	1199-11.4	60
4	1195-11.5	60
5	119,[illegible]-12.2	60
6	118,9-15,3	60
7	107,2-11,3	60
8	106,8-12.1	60

Antal normala metafaser: **Antal förändrade metafaser:**

Typ av förändring:

Datum: 161005 **Signatur:** 10

Arbetsprotokoll för FISH-analys — CEP X/Y Vysis

DXZ1 (Xp11.1-q11.1) (α satellit): Sp.Green **DYZ3 (Yp11.1-q11.1) (α satellit): Sp.Orange**

620161370 Pers.nr: 2016-09-22-U014
G4d60
71
Provt. datum: 16-09-22
Anders lindahl almedium

Diagnos:

Material: Snitt **PAD:**

Droppn. av preparat: **Sign:**

FISH analys dat: 160922 **Sign:** IÖ

Övrigt:

Analyserade interfaskärnor

Sign	2G	1G 1O					Total
IÖ	13	87					100
Summa							
Resultat %							

Analyserade metafaser **Sign:** **Sign:**

Koordinat	Resultat	Koordinat	Resultat	Koordinat	Resultat	Koordinat	Resultat

Fotograferade celler och metafaser:

Bild nr.	Koordinat	Resultat
0001	91,7-13,5	2G 6O
2	91,5-14,0	6O
3	92,0-15,8	2G
4		6O
5		6O
6	106,0-11,4	2G 6O
7	106,0-11,6	
8	105,6-11,8	2G
9	105,6-12,0	6O
10	106,2-13,0	6O

Antal normala metafaser: **Antal förändrade metafaser:**

Typ av förändring:

Datum: 161005 **Signatur:** IÖ
